# Small area population denominators for improved disease surveillance and response

**DOI:** 10.1016/j.epidem.2022.100641

**Published:** 2022-12

**Authors:** A.J. Tatem

**Affiliations:** WorldPop, School of Geography and Environmental Science, University of Southampton, UK

**Keywords:** Population mapping, Geospatial modelling, Satellite imagery, Health Information Systems, Census

## Abstract

The Covid-19 pandemic has highlighted the value of strong surveillance systems in supporting our abilities to respond rapidly and effectively in mitigating the impacts of infectious diseases. A cornerstone of such systems is basic subnational scale data on populations and their demographics, which enable the scale of outbreaks to be assessed, risk to specific groups to be determined and appropriate interventions to be designed. Ongoing weaknesses and gaps in such data have however been highlighted by the pandemic. These can include outdated or inaccurate census data and a lack of administrative and registry systems to update numbers, particularly in low and middle income settings. Efforts to design and implement globally consistent geospatial modelling methods for the production of small area demographic data that can be flexibly integrated into health-focussed surveillance and information systems have been made, but these often remain based on outdated population data or uncertain projections. In recent years, efforts have been made to capitalise on advances in computing power, satellite imagery and new forms of digital data to construct methods for estimating small area population distributions across national and regional scales in the absence of full enumeration. These are starting to be used to complement more traditional data collection approaches, especially in the delivery of health interventions, but barriers remain to their widespread adoption and use in disease surveillance and response. Here an overview of these approaches is presented, together with discussion of future directions and needs.

## Highlights

• A cornerstone of strong disease surveillance systems is basic subnational scale data on populations and their demographics.
• Population data can suffer from outdated or inaccurate census data and a lack of administrative and registry systems to update numbers, particularly in low and middle income settings.
• In recent years, efforts have been made to capitalise on advances in computing power, satellite imagery and new forms of digital data to construct methods for estimating small area population distributions across national and regional scales in the absence of full enumeration.
• Many barriers still remain to the widespread adoption and use of modelled small area population estimates in disease surveillance and response.

## 1 The value of small area demographic data for effective disease surveillance

The Covid-19 pandemic has shone a spotlight on the importance and lack of comprehensive, timely and accurate health surveillance and information systems for mitigating the impact of infectious diseases. Integrated systems that brought together timely data on cases, healthcare utilisation and deaths at small area scales, together with reliable data on underlying demographics, enabled rapid and accurate identification of outbreaks, key risk groups, scales of transmission and spread routes. These all facilitated the appropriate design of interventions and mitigation of impacts. Building such systems in the midst of a pandemic is challenging though and requires building upon existing routine systems and data collection. The Covid-19 pandemic exposed how many countries did not have such strong systems and underlying data in place (Aborode et al., 2021, Ibrahim, 2020).

Key challenges in disease surveillance and the achievement of ‘precision’ in public health, include the registration of births and deaths and tracking disease (Dowell et al., 2016). These are impacted in multiple ways by weaknesses in underlying demographic data. In terms of birth and death registration, it is hard to know whether a national deworming programme for children in one country or a vaccination programme for pertussis in another is reducing mortality when less than 5% of deaths are registered. However, even if 100% of deaths are registered, it remains challenging to both implement the programmes and place the numbers of deaths in context without reliable multi-temporal, disaggregated data on population numbers and distributions, particularly when seasonal dynamics are strong and highly mobile population groups exist (Bharti et al., 2011, Buckee et al., 2017, Wesolowski et al., 2017). Careful surveillance can guide public health in a country (Dowell et al., 2016), but improving detection and measurement of the numerator without attention to the denominator however risks providing an inaccurate picture. Analyses in Namibia showed that improved quantification of denominator populations changed malaria incidence measures by more than 30% (Zu Erbach-Schoenberg et al., 2016). Moreover, by pairing just a small number of physical autopsies with verbal autopsies on the same deaths, the much larger number of verbal autopsies can be calibrated - but the verbal autopsy data are often drawn from surveys built on static and outdated sample frames (Carr-Hill, 2013, Thomson et al., 2020) and again it remains challenging to place outputs in context in settings where denominators are uncertain. The reliance on static and aging figures for denominators leads to the common occurrence of 200% vaccination rates (Cutts et al., 2021).

The collection and maintenance of timely and accurate small area data on population distributions, demographics and dynamics represents a challenge across the World. In many countries, Covid has exposed a lack of registry systems for recording cases, deaths, but also a lack of timely and reliable data on denominators. Moreover, Covid itself has disrupted the improvement of this situation. Seventy-three percent of National Statistical Offices (NSOs) had a Population and Housing Census planned in 2020 or 2021 before the pandemic hit (Contreras-Gonzalez et al., 2020, UNFPA, 2020a). In the low and lower-middle income group, 68% of the NSOs that were planning a census had to postpone it (United Nations, 2020).

The value of small area demographic data remain clear and the Covid-19 pandemic demonstrated key applications of modelled geospatial datasets. They formed the demographic basis for some of the most high profile and influential covid transmission models used to guide policy (Imperial College, 2022, Institute for Health Metrics and Evaluation, 2022), and are now built into health information system software (University of Oslo, 2022). They were also used in the construction of microplans for the delivery of covid vaccines and in reacting and assessing responses to outbreak and mitigation measures (e.g (GRID3, 2021a, Shepherd et al., 2021).). These applications demonstrate a growing acceptance and use of modelled population datasets built upon geospatial data from satellite imagery, GPS mapping and mobile data to fill gaps in small area demographic data availability. Nevertheless, the construction of such datasets remains an area of research, with substantial uncertainties remaining and differing inputs and approaches leading to large variations in output estimates.

### 1.1 Modelled small area pop estimates

Ideally, every country would have systems such as those in Nordic countries, where integrated registries and administrative data collection systems enable the production of timely small area data on population distributions and demographics, without the need for costly decennial national population and housing censuses (UNECE, 2018). While more countries move towards developing such systems, many are far behind, with the implementation of a national census every ten years still remaining a challenge. Even where these are implemented in a robust and rigorous way, demographic changes during intercensal periods can make the data rapidly outdated, particularly at small area scales where changes are harder to forecast. In some countries, registry and administrative systems can fill these gaps, but these can often be incomplete and inaccurate, especially in many low income settings. Rolling surveys are another solution that some countries have adopted to capture changes between censuses at relatively low costs, such as the US Census Bureau’s American Community Survey (Census, 2022), but these are also lacking across low income settings. Spatial modelling approaches that aim to address some of these challenges through use of satellite imagery and other geospatial data to capture small area changes occurring over relatively short timescales compares to the decennial census have therefore become more prominent in recent decades (UNFPA, 2020b).

In the absence of publicly available small area data from censuses, or in the absence of consistent data between countries and across continents, or in the absence of any recent and reliable data at all, spatial modelling approaches aim to fill gaps. Since the 1990s (Tobler et al., 1997), a major focus has been on so-called ‘top-down’ disaggregation, whereby large area census data, or projections matched to relevant administrative/enumeration boundaries are disaggregated to grid squares, maintaining counts at original units (‘mass-preserving’) and estimating distributions within these units (Tobler, 1979, Leyk et al., 2019). The ongoing global assembly of such unit-based count data (CIESIN, 2018) has meant that different approaches to disaggregation have been explored and applied over recent years. These have been driven by the availability of settlement maps, relevant geospatial covariates and computing power among other factors, as well as intended user needs. Some have maintained the simplicity of simply spreading available population data equally over a grid, some have allocated population counts to mapped settlements, while others have built more complex models that use a set of geospatial covariates to try and capture variations within settlements and across countries. The focus of this perspectives piece is not to provide a comprehensive review of these approaches and resultant datasets, but readers interested in more background should refer to Leyk et al. (2019). for a recent review.

**Fig. 1:**
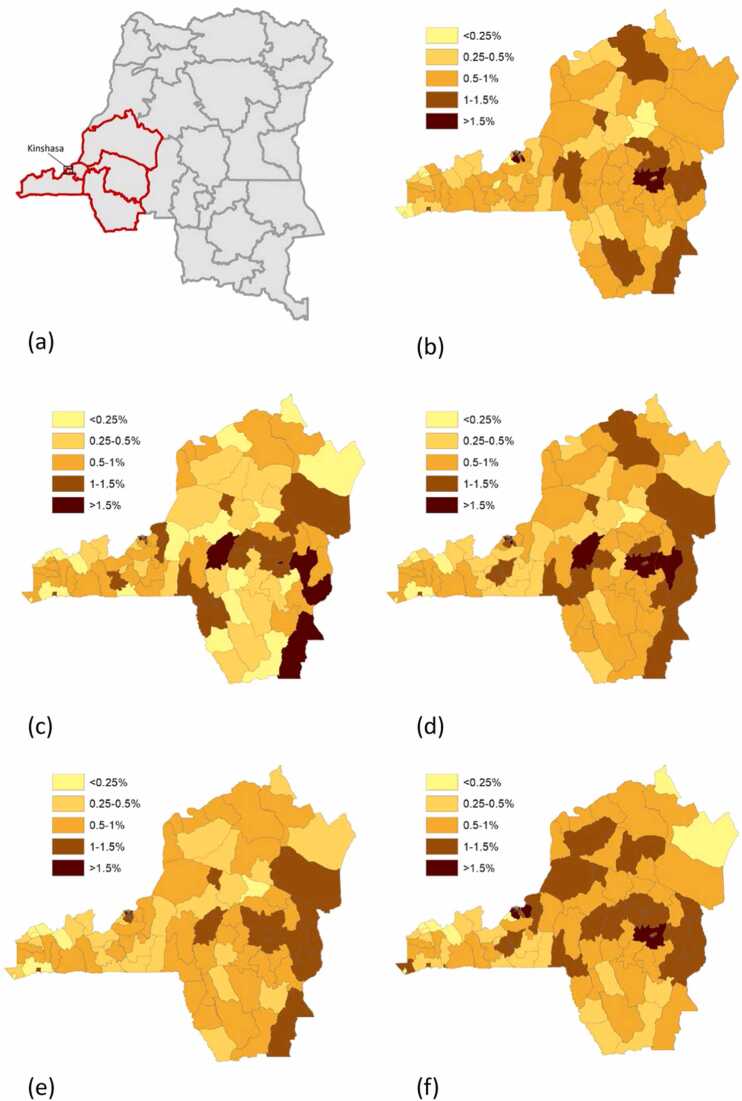
Maps for the region encompassing the five most westerly provinces of the Democratic Republic of the Congo (Kinshasa, Kongo-Central, Kwango, Kwilu, and Mai-Ndombe, as shown in (a)), showing proportions of the total population of the region estimated to be in each health zone for a set of commonly used open gridded population datasets: (b) Gridded Population of the World version 4 (GPWv4) (CIESIN, 2018), (c) GHS Population grid (Florczyk et al., 2019), (d) Meta Data for Good High resolution population density maps (Meta Data for Good, 2022), (e) WorldPop global constrained top down estimates (Bondarenko, 2020), (f) WorldPop/GRID3 bottom up population estimates (Boo et al., 2020).

**Fig. 2:**
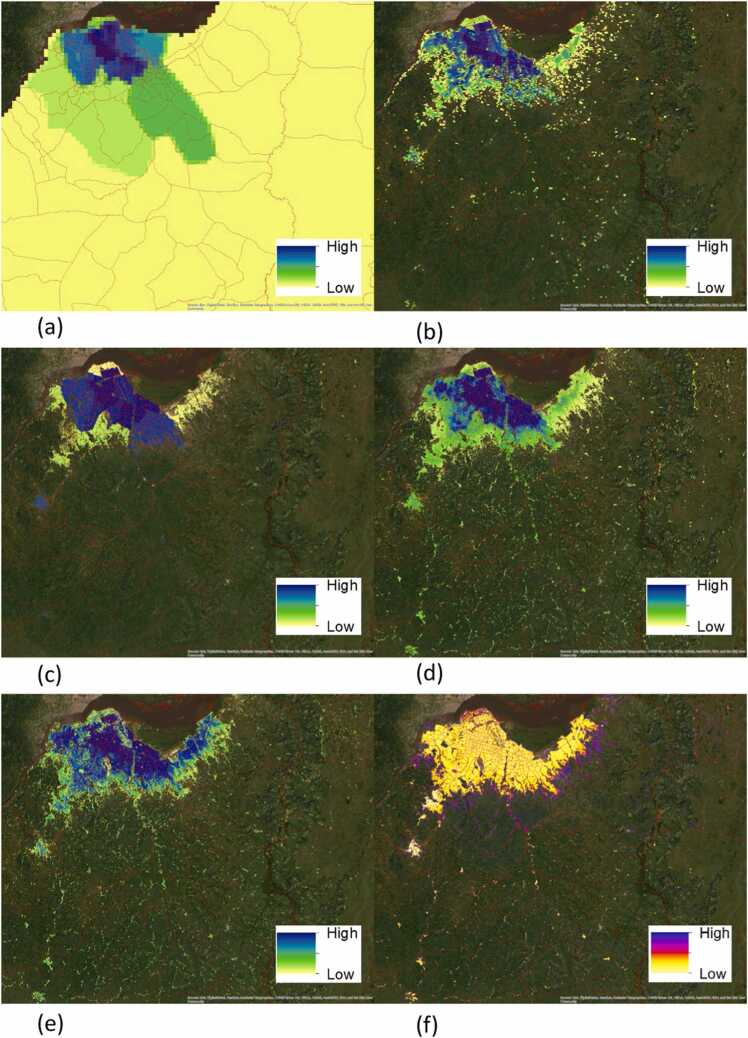
Gridded population estimates from the same datasets as Fig. 1 for Kinshasa and surrounding area in the Democratic Republic of the Congo, with health areas overlaid. The extent of the area shown is highlighted in the black box in Fig. 1(a). Each dataset has been displayed using a scale of 20 quantiles within the area shown to highlight the inherent population distribution patterns. (a) Gridded Population of the World version 4 (GPWv4) (CIESIN, 2018), (b) GHS Population grid (Florczyk et al., 2019), (c) Meta Data for Good High resolution population density maps (Meta Data for Good, 2022), (d) WorldPop global constrained top down estimates (Bondarenko, 2020), (e) WorldPop/GRID3 bottom up population estimates (Boo et al., 2020), (f) uncertainty of WorldPop/GRID3 bottom up population estimates, measured as the difference between the upper and lower 95% credible intervals of the posterior prediction divided by the mean of the posterior prediction.

The variety of ‘top down’ modelling approaches results in differing disaggregations of the same aggregate population count data. Using the example of the five most westerly provinces of the Democratic Republic of the Congo (DRC) that encompass Kinshasa and its surrounding region, Fig. 1 highlights how different a selection of commonly used open model estimates can be at the scale of health zones. These will in turn result in differing surveillance indicators, denominators for health metrics (Nilsen et al., 2021), and target populations for interventions. Fig. 2a-d shows the relative patterns of estimated population distributions in each dataset at the grid square scale for Kinshasa and its immediate surrounding area with health area boundaries overlaid. While DRC represents an extreme example, having not conducted a national census since 1984, and the population estimates input to the models are for large administrative units, the figures highlight how variations in top-down modelling methods can result in substantial differences in predictions. An obvious question when presented with such differing estimates of population distribution is ‘which is right?’. This is often a challenge to assess, as where detailed and recent population data exists, often it is the input to the models, leaving a lack of independent data to compare against. Studies using detailed census data (Bustos et al., 2020, Chen et al., 2020, Fries et al., 2021, Yin et al., 2021), as well as cross-validation (Reed et al., 2018, Stevens et al., 2015, Stevens et al., 2020) have tried to assess how well different models replicate population numbers and distributions at the scale of available data, and unsurprisingly those more complex models using detailed settlement mapping and range of covariates tend to do best. However, multiple trade-offs exist in the production of such datasets that depend on aspects such as input data availability, geographical extent, temporal range, spatial resolution, intended use and user needs. For instance, a more complex model may produce more accurate outputs, but the production process can be more challenging to communicate to users. A dataset that estimates population distributions over multiple years will necessarily have to compromise on the quality and amount of data for older time periods, and a high spatial resolution dataset can be more difficult to process and incorporate into surveillance systems than a coarser scale one. Each model and dataset has often been designed with a different purpose in mind and have different types of uncertainty and variations inherent within them, which can often make them not directly comparable.

A bigger issue than whether one model or another more accurately distributes the population counts from administrative unit to grid square scale, is the fact that the population counts themselves can be uncertain and inaccurate. The age, scale, type and quality of input population count data being disaggregated within top-down models is information that has often been poorly communicated, understood and addressed. While gridded outputs therefore tend to look similar between countries, there can be substantial variations in the accuracy of population estimates. The differences between the DRC and nearby Malawi are illustrative, with input population count data for DRC coming from uncertain projections from the 1984 census and an average unit size of 12,476 km2, while the data for Malawi come from their 2018 census, with an average unit size of just 9.4 km2. In these two settings, the choice of modelling approach has a significantly larger impact for the DRC, where population counts for massively larger units are being disaggregated to the same size grid squares and the differences seen in Fig. 2a-d would be less apparent for Malawi. Nevertheless, as highlighted above, even in settings with high quality regular censuses with data available mapped to small units, the processes of migration, displacement, urbanization and heterogeneous fertility and mortality can make these data quickly outdated and are hard to accurately forecast at small area scales (Wilson et al., 2021), resulting in potentially major impacts on reliable surveillance and health metrics (Tatem, 2014).

The issues outlined above have lead to the rise of census-independent small area pop estimation methods (Francoise, 1990, Harvey, 2002, Hillson et al., 2015, Leasure et al., 2020, Wardrop et al., 2018, Weber et al., 2018). These ‘bottom-up’ modelling methods typically rely on complete counts of population within small defined areas that can come from bespoke field surveys, listings from household surveys, or the results of rolling or partial censuses. Statistical models are then used to link these enumeration data to spatial covariate data, with full coverage over the regions of interest to predict population numbers in unsampled locations (UNFPA, 2020b, Wardrop et al., 2018). Often these are Bayesian models built to try and capture as many forms of measurable uncertainty and error in inputs and the modelling process as possible, and propagate them through to output predictions. The models produce full posterior prediction outputs for each grid square, meaning they can be summarised to produce most likely estimates, but also measures of uncertainty at differing levels and spatial scales. Recent examples include the development of models to estimate population counts in areas that could not be enumerated in national census efforts in countries such as Burkina Faso, Afghanistan and Colombia (Darin et al., 2022, Sanchez-Cespedes et al., 2021, WorldPop Mapping, 2021). In Zambia, models were developed to make use of household listings from recent survey data as training data to construct national small area estimates that formed the basis for census planning and health intervention delivery (Dooley et al., 2021, GRID3, 2021b, WorldPop, 2021a). Moreover, in settings where existing census data are outdated, bespoke field surveys were undertaken to obtain recent sample enumeration data for constructing modelled estimates in Nigeria, South Sudan and DRC (Leasure et al., 2020, Boo, 2022, UNFPA South Sudan Population, 2021). The outputs of the bottom-up modelling efforts in DRC are presented in the same way as those from the multiple top-down models in Fig. 1f and 2e, but with the addition of a measure of prediction uncertainty mapped in Fig. 2f (Boo et al., 2022). Given the significantly different input data and approach to production of the estimates, it is no surprise to see more differences in estimated patterns of population distributions in the figures.

Are the bespoke bottom-up population estimation models producing more accurate estimates of small area population numbers than the top-down approaches? This is difficult to assess and remains context and location-specific, but there is growing evidence and many reasons to believe that the estimates are more reliable. While sample sizes are often small, building models upon recent enumeration data, rather than linear projections from census baselines many decades ago in settings where massive changes have occurred provides more confidence in outputs (Wardrop et al., 2018). A growing amount of anecdotal and quantitative feedback from field teams and national statistical offices on the accuracy of estimates adds to statistical evidence from model cross-validation, as well as assessments on the use of data in deriving metrics or in health delivery campaigns adds to this (Nilsen et al., 2021, Leasure et al., 2020, GRID3, 2021b, Boo, 2022, GRID3, 2020, Thomson et al., 2021, Ali et al., 2020). Moreover, the explicit measurement and communication of uncertainty in predicted population estimates provides users with quantitative insights on where confidence in predictions is higher or lower, taking small area population estimates a step forward beyond the opacity of many top-down model outputs (Leasure et al., 2020).

### 1.2 The future for small area population data

Geospatial modelling approaches have made great advances in supporting the estimation of population numbers at small area scales. They should not be seen however as a replacement for censuses, surveys and systems of enumeration. These ensure that people are counted and are the source of a wealth of additional data that cannot be accurately estimated from models based on satellite imagery and other forms of geospatial data. Nevertheless, the challenges that physical enumeration of populations poses should be recognised (UNFPA, 2020b). National population and housing censuses are typically the largest peace-time operations that countries undertake, and the expense, complex logistics and disrupting factors, such as conflict or covid, mean that full enumeration is not always possible. This in turn results in a lack of reliable and timely data that form a key component of disease surveillance systems. Recent years have seen a growing use of modelled population data by ministries of health, national statistics offices and international agencies, in particular estimates produced from bottom-up models (Wardrop et al., 2018). As well as being used for health-related applications such as vaccination or bednet distribution campaigns (GRID3, 2021b, Ali et al., 2020), the value of these estimates in supporting the census process (UNFPA, 2020b) or collection of survey data (Thomson et al., 2020, Qader et al., 2021, Qader et al., 2020, Thomson et al., 2017) has been shown.

Many challenges remain to be addressed in capturing accurate population numbers at small area scales through both enumeration and modelling approaches, but ongoing research points towards potential solutions. Multiple innovations in data collection and sample design are pushing forward the ability to directly enumerate populations and capture data from those that can be hard to reach (Hoogeveen and Pape, 2020; Tomaselli et al., 2021). These can provide valuable data to complement and improve upon traditional approaches to enumeration, as well as form the basis for the geospatial modelling efforts that are the focus here. Substantial variations in population densities and land uses over small spatial scales make accurately estimating and mapping populations within urban areas a difficult exercise, but the automated ability to accurately map building footprints from recent satellite imagery is helping to quantify some of this variability (Boo et al., 2022). The further processing of these building datasets to map neighbourhood types (Jochem et al., 2020) and categorize residential status (Sturrock et al., 2018) is supporting refinements to urban population modelling (WorldPop, 2021b). Moreover, the development of approaches for estimating building heights and volumes from satellites (e.g (Esch et al., 2022).) presents opportunities to account for high rise residential or commercial buildings in estimation modelling. Geostatistical modelling from GPS-located survey data also offer solutions for the small area mapping of population demographics to move beyond large area summaries or outdated census data (e.g (Alegana et al., 2015).).

The dynamics of urban populations present challenges, with constant changes in densities each day, week and season, and urbanization trends changing the shape and extent of settlements rapidly. Here again, new forms of geospatial digital datasets offer possibilities to capture and quantify such changes that would be costly to measure with surveys or full enumeration. These include the use of mobile phone call records (Bergroth et al., 2022, Deville et al., 2014), satellite-based measures (Bharti et al., 2016) and models that integrate multiple forms of spatially referenced data (Martin et al., 2015). Subnational changes in population distributions induced by migration and displacement have been shown to be reliably captured by models driven by mobile phone record data (Lai et al., 2019, Bengtsson et al., 2011), and approaches for incorporating such flow data into small area population estimation models continue to be explored (Dooley et al., 2020). Improved understanding of the processes and dynamics of population changes at small area scales in turn offer the potential for improved forecasting (Wilson et al., 2021).

Ultimately, national statistical offices hold the responsibility for the production and maintenance of the official population data that feed into disease surveillance and health information systems. Many have invested in geospatial, geostatistical and data science skills to capitalise on advances in small area population data production. Across low and middle income regions, resources for such investment are often limited, and therefore challenges exist in abilities to adopt, develop and integrate new geospatial methods to complement more traditional enumeration approaches. Official population data underlie governance and can be highly sensitive since they determine allocations of resources, representations in parliaments and delivery of services. It is generally insufficient therefore for models developed and implemented elsewhere to be handed over to statistical offices. Country ownership and the ability of statistical offices to explain and defend methods adopted in the production of small area population data becomes vital for acceptance and use. This will remain a substantial barrier to the use of geospatial modelling to complement more traditional data collection, but the examples highlighted above from countries such as Burkina Faso, Colombia, Nigeria, DRC, Afghanistan and Zambia show how co-development and country ownership are beginning to address these challenges, providing data that can then be used by Ministries of Health as an important component of surveillance and healthcare delivery.

The Covid pandemic has necessitated the acceleration of vaccines and treatment development, of the science of disease modelling, and of the shape and scale of infectious disease surveillance. The disruption that the pandemic has caused to traditional methods of population enumeration in some countries (Contreras-Gonzalez et al., 2020, UNFPA, 2020a) may also necessitate the accelerated development and adoption of modelling approaches to fill gaps. Moreover, increased demands for timely and reliable small area denominator data to support the needs of expanded and reshaped disease surveillance systems will bring extra demands. Multiple types of denominator data exist, each with their own set of strengths, different sorts of uncertainty and different levels of accuracy. Clearly reporting the denominator data used, its source and features, as well as quantifying uncertainties should ideally be a goal for analyses and reports based upon them. Geospatial modelling can become an important tool in integrated approaches to the production of small area population data. For example, where a survey or new census is being planned, modelled estimates can provide a sample frame where previous census-based data are outdated, and in turn, the data collected using the new frame can be used to improve and update the modelled estimates for future use. These complimentary activities offer the possibility of moving towards a kind of ‘living’ census, something that is currently only a reality in countries with strong registry-based systems. In the meantime, geospatial modelling approaches provide a way to compliment ongoing data collection efforts to provide more timely and finer scale estimates, either through the ‘top-down’ spatial disaggregation of projections to small areas, or the ‘bottom-up’ estimation of population numbers from sample data into unenumerated areas. Having access to timely, reliable population data at small area scales that is able to be regularly updated should ideally be a target for the World to work towards to ensure appropriate, effective and efficient responses when the next outbreak or pandemic arrives.

## CRediT authorship contribution statement

AJT conceptualised the paper, and undertook the analyses and writing.

## Declarations of interest

None.
